# Human Performance Analysis of Processes for Retrieving Beidou Satellite Navigation System During Breakdown

**DOI:** 10.3389/fpsyg.2020.00292

**Published:** 2020-02-21

**Authors:** Mo Wu, Liang Zhang, Wen-Chin Li, Lingyun Wan, Ning Lu, Jingyu Zhang

**Affiliations:** ^1^CAS Key Laboratory of Behavioral Science, Institute of Psychology, Beijing, China; ^2^Department of Psychology, University of the Chinese Academy of Sciences, Beijing, China; ^3^Beijing Satellite Navigation Center, Beijing, China; ^4^Safety and Accident Investigation Centre, Cranfield University, Cranfield, United Kingdom; ^5^School of Psychology and Cognitive Sciences, Peking University, Beijing, China

**Keywords:** satellite navigation system, fault handling, task complexity, time of day, shift handover, team skill level, incident-based analysis

## Abstract

Satellite navigation systems provide continuous, timely, and accurate signals of location, speed, and time to users all over the world. Although the running of these systems has become highly automated, the human operator is still vital for its continued operation, especially when certain equipment failures occur. In this paper, we examined 180 incidents of one particular type of equipment failure and the whole recovery process as recorded in the log files from a ground control center of the Beidou satellite navigation system. We extracted the information, including the technical description of the failure, the time when the fault occurred, the full recovery time, and the demographic information of the team members on the shift responsible for responding to the failure. We then transformed these information into the cognitive complexity of the task, time of day, shift handover period, and team skill composition. Multiple regression analysis showed that task complexity and shift handover were key predictors of recovery time. Time of day also influenced the recovery time, during midnight to 4 a.m., operators made longer responses. We also found that the fault handling processes could be improved if the team’s most adept member is more skillful at that role than in other teams. We discussed the theoretical and practical implication of this study.

## Introduction

Satellite navigation systems play an important role in modern society. For example, the well-established US-owned Global Positioning System (GPS) provided $ 68.7 billion economic benefits in 2013, making up 0.4% of U.S. GDP (Leveson, 2015). Satellite navigation systems consist of 1000s of instruments, and each instrument has different functions. If a certain instrumental failure occurs, the system may not be able to provide its service in a normal manner. In that case, quick recovery is key for system effectiveness. Otherwise, it can result in serious disturbance to its users all over the globe (e.g., the EU-owned GALILEO system suffered a mass outage on 12 July 2019 because of the Precise Time Facility fault, which took a week to recover). Although automation has been widely introduced to current satellite systems, remote human operation from a ground control center is still crucial, especially in responding to certain equipment failures. Operators are required to provide immediate and correct responses to detect, diagnose and resolve the fault, but their performance is heavily influenced by certain human factor variables which are also important for human-computer interface design, training, and management.

Human factors in the control of space-based systems were studied many years ago. For example, Kuhr and Tobias (1974) studied human factors engineering in the design of a satellite communication system. They provided the principles and constraints of the design and corresponding verifications in some control terminals. To meet the requirement of advanced automation, human factors were introduced to design NASA’s near-earth satellite control center (Mitchell, 1982; Van Balen and Mitchell, 1983). They provided a data-gathering method and introduced a methodology for human factors analysis and design. In an empirical study, Charlton (1992) investigated how self-reported human factor variables (e.g., usefulness of documentation, display layout, workload, noise level, fatigue, etc.) could influence the operation time in space control centers. Neville et al. (2003) studied the design of a toolset for operators in changing satellite operations. They described work-centered user interfaces design principles and provided a satellite viewing tool as an example. However, these studies are quite old, and their conclusions were based on a pre-operation evaluation, rather than on empirical operational data.

Although direct evidence on factors that influence the operation of this special task is limited, studies on other process control operations with similar working requirements can provide valuable information. The fault diagnosis and resolution process has been studied intensively in the nuclear power plant, air traffic control (Langer and Braithwaite, 2016; Trapsilawati et al., 2016; Borst et al., 2017), submarine (Roberts et al., 2017), and many other backgrounds (Vicente et al., 2004; Wickens et al., 2009; Sebok and Wickens, 2016). Operator performance of safety-critical and highly automated environments monitoring has also been studied (Funke et al., 2016; Cymek, 2018). Previous studies have shown that several key factors can influence operator performance during fault handling, including task complexity (Liu and Li, 2012), positional knowledge and skill (Littlepage et al., 2016), and environmental stress (Martin et al., 2019), etc. However, no direct empirical research has been reported on the fault handling process of satellite control. To fill these research gaps, we examined how task, person and environmental characteristics jointly influenced the fault handling process of the satellite navigation system based on actual event data. In the following paragraphs, we described the operation requirements and processes of the Beidou Navigation Satellite System; then, we reviewed the literature on important factors that might influence the process by considering studies from other process-control operations; finally, we provided a research scheme on how to test the influence of each factor using actual event records.

### The System Control and a Typical Fault Handling Process of Beidou Navigation Satellite System

Beidou Navigation Satellite System (BDS) is one of the four navigation systems in the world, the other three are GPS of the United States, GLONASS of Russia and GALILEO of the European Union. Constructed and operated by China, BDS provides all-time, all-weather and high-accuracy positioning, navigation, and timing services to global users.

The operational control system of BDS is composed of two independent departments, navigation service control center (NSCC) and satellite platform control center (SPCC). The two departments account for different functions. The major work of NSCC operators is to monitor the functioning of the navigational information system installed on the satellite (can be considered as an application software system). The operators at SPCC, on the other hand, are controlling all on-board instruments and the operating system of the satellites.

**FIGURE 1:**
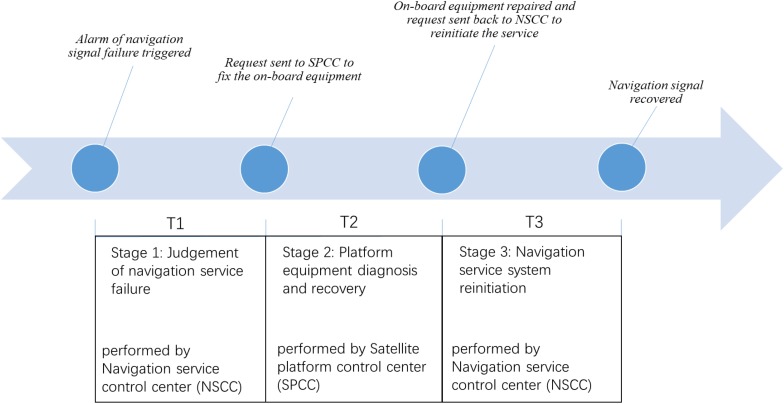
The four critical events (the circles) and the three stages of operations of an instrument failure handling process.

In the NSCC, operators work in teams to monitor the navigational information system on a 24-h basis, and one of their major tasks is to detect, diagnose, and resolve possible instrument/system failures. When any on-board equipment failure occurs, the system will alarm, the operator team then detects the alarm and makes a preliminary diagnosis (stage 1). If they find it is a satellite equipment failure, they will request SPCC to conduct the on-board checking and equipment recovery (stage 2). After the on-board equipment is recovered, the NSCC will receive the feedback from the SPCC and start to make a series of operations to re-initiate the navigational information system (stage 3). The whole instrument failure handling process ends when the navigation signal received from satellite returns to normal. This process is illustrated in Figure 1. During the whole process, all the important time points are recorded, which provided valuable information on task completion times of all three stages (T1, T2, and T3) for human factor researchers.

### Key Human Factors That May Influence the Recovery Process of the Satellite Navigation System

Given that the equipment failure incidents analyzed in this paper were all resolved, we took the task completion time as the most important evaluation index of the handling process. The key human factors can impact on either the task requirements or the operator teams’ capacity, and the completion time may be prolonged when their capacity or capability fails to meet the requirments.

#### Task Complexity

Task complexity is an intrinsic task characteristic which evaluates the structure and requirement of a task (Wood, 1986; Gonzalez et al., 2005; Liu and Li, 2012). From an objective perspective, task complexity is often operationalized using standard operating procedures, necessary coordination loops, and dynamic information processing (Wood, 1986). This objective task complexity is independent of task executors. Although increased task complexity may sometimes increase operator performance when the baseline tasks are too simple and boring (Pepinsky et al., 1960; Wood, 1986; Tan et al., 2002), higher task complexity is often related to lower operator performance (Liu and Li, 2011). This negative effect is particularly common for failure diagnosis and resolution in a highly dynamic and complex automated systems (Mumaw et al., 2000; Vicente et al., 2001). For example, Molloy and Parasuraman (1996) found that in a simulated flight task, participants performed worse in detecting the failures when the task was more complex. Similarly, Mumaw et al. (2000) found that more complex situations could increase the difficulty to detect and resolve abnormal events in nuclear power plants.

In our study, we focus on operators’ performance in fault diagnosis and resolution for the highly dynamic and complex Beidou satellite navigation system. Task complexity would be determined by system and fault characteristics regardless of operators’ characteristics. Conflicts between high cognitive complexity and limited information processing ability may lead to longer operating times. Therefore, we assumed that there is a positive relationship between task complexity and task completion time (hypothesis H1).

#### Shift Handover

Shift handover occurs when two shifts rotate. The goal of shift handover is to effectively communicate task-related information across shifts, thereby guaranteeing “continuity of safe and effective working” (Brazier and Sedgwick, 2011). However, there is often insufficient or erroneous communication during this period, which may lead to near-misses or even accidents. Accident investigations found that handover problems were among the root causes of many critical events. For example, the incomplete handover of fault handling between two shifts of the control room was a leading cause behind the Piper Alpha incident (Kontogiannis et al., 2000). Doytchev and Szwillus (2009) also pointed out that incomplete information handover was an important reason for a basement flood accident of a nuclear power plant in Bulgaria. Researchers using a larger data set of incidents also found a link between shift handover and accidents. For example, deficient handover can increase transmission errors and related accidents in the offshore oil industry (Gordon, 1998). Similarly, incomplete or non-handover can result in patient safety issues in medical settings (Pezzolesi et al., 2010, 2012; Riesenberg, 2012). Siemsen et al. (2012) also found evidence that shift handover is one of the factors affecting task performance in nuclear power plants control rooms.

Through the literature review, it is clear that insufficient handover may lead to negative effects on tasks. In practical terms, the incoming team may not have a comprehensive grasp of the system state, resulting in failure to complete or repetition of certain steps during the fault handling process, thus increasing the processing time. Indeed, even if the handover is sufficient, making a handover itself can increase the cognitive and communicative burden on both shifts during the handover period, which may occupy the limited cognitive resource originally assigned to the task. In this way, we proposed that when an unexpected fault occurs during the shift handover, the fault handling process might be affected, and the task completion time would be prolonged (H2).

#### Time of Day

Because many human psychological processes and mental functions are influenced by circadian rhythms (Folkard, 1983; Campbell, 1992), task performance of the satellite fault handling process can also be affected by the time of day.

Circadian rhythms are changes in physical and mental functions, from nervous system activity to hormone production, following a 24-h cycle. Although these rhythms vary from individual to individual, for most people, the lowest physiological activity occurs around 4:00 to 6:00 a.m. (Monk et al., 1997). Task performance is also influenced by these rhythms. Generally, the highest performance level occurs in the morning, and the lowest occurs from midnight to 6 a.m., which is related to the law of human body temperature cycle (Colquhoun, 1972; Dongen and Dinges, 2000).

In the fault handling task of the satellite navigation system studied in this paper, the fault can occur at any time of day, in this way, providing a natural experiment to examine whether the time of day can influence the task performance of operators. It was assumed that the time of day might affect the task completion time of the fault handling process (hypothesis H3).

#### Team Skill Composition

Since the satellite control task is performed by operator teams, the characteristics of the team can also play a role in influencing the performance. In the IMOI model that guides most team research, team composition, especially the composition of team members’ capabilities, is an important input variable (Ilgen et al., 2005; Mathieu et al., 2013). It is found that the ability level, competence, and mental model of team members have a significant impact on team performance (Kearney et al., 2009).

To quantify team composition, the average level is mostly used. For example, Resick et al. (2010) found that the averaged team cognitive ability was most predictive of team performance. Zhang et al. (2013) also found that the average mental ability score of control room operators in nuclear power plants was significantly related to team performance. Given most team tasks require a collaboration of all team members, it is not surprising that the mean skill level, which best reflects the team as a whole, can have the largest effect. In certain teams with specific team structure and task requirement, however, other quantification methods (e.g., the highest value) can also predict team performance (Devine and Philips, 2001). For example, if the team task has a clear division of labor and the fulfillment of the task relies heavily on the expertise possessed by the most specialized team members, then the highest skill level of the team might also be related to the team performance (Bell, 2007).

In handling the failure of a satellite navigation system, operators in a team have to work closely with each other. Meanwhile, since the work is highly specialized, they also have a clear division of labor, and each member must use their expertise to handle relevant sub-tasks when needed. In this way, both the averaged team skill level and the highest skill level can influence team performance. As a result, we made the following hypotheses.

Hypothesis H4-a: The average team skill level has a positive impact on task performance;

Hypothesis H4-b: The highest team skill level has a positive impact on task performance.

### The Present Study

In this study, we would fully utilize actual event log records to research the failure process of satellite navigation instrument, hoping to examine how task complexity, shift handover, time of day, and team skill composition jointly influence instrument failure recovery process. While the detailed description could be found in the method section, two important aspects must be addressed here, as they were related to the general expectations of our research. First, we would analyze the task completion times at two different operation centers (T1 and T3 at NSCC, T2 at SPCC), but we only collected the shift schedule and team composition information from NSCC, so these two factors would be more likely to exert influence on T1 and T3. Second, the information was collected from actual log records which were written by the operators and only the minute level information was recorded, so the reliability of the models would be undermined when the total lengths of the time interval were shorter (T1).

## Materials and Methods

### The System

The task of the research is defined as diagnosing and processing satellite navigation instrument failure. One certain category of events, Instrument N^1^ failure, was extracted and analyzed in this study. Satellite navigation instrument failure usually results in unavailability of the satellite, which may reduce the navigation precision of users. Accordingly, a quick solution for instrument failure is very important for the navigation satellite system.

Instrument N failure was chosen in our study for two reasons. First, this failure is one of the typical failures of a satellite navigation system. According to the event log of the system, Instrument N failure is responsible for about 18.1% of all failures as the largest category of all. Second, its diagnosis and recovery process follows a clear hierarchy. When Instrument N failure occurs, operators perform a diagnosis and a series of fault handling. The whole process of fault handling is divided into two parts, on-board operation and service control operation. On-board operation is referring to the on-board functional recovery, including diagnosis of the failure and corresponding operations, while service control operation is referring to the navigation business recovery, including instrument operating, information creating, and feedback confirming. Most of the operational steps are executed sequentially.

The Instrument N involves two modules: module A and module B. As module A is tightly associated with the navigation precision of users, we chose the module A failure as the target of our research. We have taken an approach of using actual data in the course of operation and maintenance of the NSCC. The task of the research was defined as diagnosing and disposing of the Instrument N failure.

### Data

We were provided with actual data from the NSCC, which is responsible for the management of BDS ground station. Such data included properties of the fault handling process. Failures occurred randomly and the operators on duty recorded the key information. We were provided with 180 Instrument N fault handling records, from a total of 635 records over 16 months of operation, which included failure time, failure circumstances, length of handling time, and operator team information. The failure circumstances contained failure module and satellite visibility information (Chen et al., 2016). Length of handling time contained judgment time (T1), satellite platform diagnosis and recovery time (T2) and navigation service system re-initiation time (T3).

Further analysis showed that these kind of events could happen at any time, regardless of the time of a day, or any particular previous operation. In this way, it provided a very good natural experiment to test our key hypotheses.

### Dependent Variables

Three dependent variables were used. Judgment time (T1) is defined as the length of time for the operators of NSCC to make a quick judgment to locate the failure to be Instrument N on the satellite (*M* = 3.02 min, *SD* = 2.55). Satellite platform diagnosis and recovery time (T2) is defined as the length of time used by the SPCC operators to diagnose and recover the on-board equipment (*M* = 22.59 min, *SD* = 18.66). Navigation service system re-initiation time (T3) is defined as the length of time used by NSCC operators to make a series of operations to recover the navigation information system (*M* = 52.71 min, *SD* = 29.97). The beginning and the end of each period were determined using the four events illustrated in Figure 1. To reiterate, the entries in the log files (for example, entry 1: alarm detected at 4:43 a.m.; entry 2: request send to SPCC at 4: 47 a.m.) were all written by the operators and they only recorded minute level information, so the time interval (in this example, T1 = 4:47 a.m.- 4:43 a.m. = 4 min) were only accurate to minute. While this measurement error is mostly random which can be reduced by a large sample, it is worth noting that its influence might be more severe for models in predicting T1, as compared with T2 and T3, since the former is much shorter.

### Independent Variables

We totally defined 15 independent variables based on the four factors, task complexity, time of day, shift handover, and team skill level, that were expected to affect the performance.

#### Task Complexity

The evaluation of task complexity was based on the *post hoc* analysis of the actual equipment conditions. Based on expert interview and task analysis, task complexity was determined by two objective factors: whether the two sub-modules in the instrument failed simultaneously and whether the satellite was observable by the Beijing ground station accessing the satellite signal directly, rather than from other ground stations (Wang et al., 2019). In the Instrument N failure incidents, two sub-modules can fail simultaneously, and the diagnosis and recovery becomes more difficult under that circumstance. Besides, when the satellite is invisible, the operators need to adopt more procedural steps, including using information from other ground stations and satellite-satellite communication, to diagnose the failure. Therefore, task complexity increases. According to the factors above, task complexity is divided into three levels. Definition of task complexity is detailed:

Level 1: only one module fails, and the satellite is within direct observable range.

Level 2: both modules fail, while the satellite is within direct observable range.

or: only one module fails, and the satellite is outside direct observable range.

Level 3: both modules fail, and the satellite is outside direct observable range.

#### Time of Day

A series of dummy variables were created to account for the time of day in the regression analysis to rule out certain non-linear relationships. In this study, 12 2-h periods were used. The first interval is 0:00–2:00, and the 12th is 22:00–24:00. We used the interval 8:00–10:00 as the reference interval because both T2 and T3 were the shortest in that period. Therefore, 11 dummy variables were created to represent this factor.

#### Shift Handover Period

The shift handover period was defined as “1” if it fell in the specified period (30 min before or after the shift handover time at 8:30 and 16:30, that is, 8:00–9:00 and 16:00–17:00, respectively) and as “0” if it was another time. To note, since only the shift schedule of the NSCC operators were available, the shift handover variable was more likely to be predictive T1 and T3, rather than T2 (which was performed by SPCC operators).

#### Team Skill Composition

First, the skill levels of three NSCC operators who were on-site during the incident were extracted. The data in the event log was recorded strictly according to the duty group at the time of failure. Their relative expertise in handling the Instrument N failure was evaluated on a 1 (just qualified) to 3 (very competent) scale given their experience and majored discipline by three experts and managers, and the inter-rater reliability of the evaluation was 0.94. Second, the maximum skill level and average skill level were defined as two independent variables and computed for each event. To note, since only the information of the NSCC operators were available, the skill level variables were more likely to be predictive T1 and T3, rather than T2 (which was performed by SPCC operators).

In addition to being on duty in the control room, operators were also engaged in daily work during their daily working hours, such as the maintenance, modification, and upgrading of the system. Daily work is classified into six majors: satellite-ground collaboration, measurement and communication, information processing, time unification, performance monitoring, planning and control. The six majors are divided into three levels according to the degree of correlation with equipment N failure.

Level 1: time unification.

Level 2: measurement and communication, information processing.

Level 3: satellite-ground collaboration, performance monitoring, planning, and control.

## Results

### Regression Analysis

We conducted a series of hierarchical regression analyses to predict all three times (T1, T2, and T3) using task complexity, time of day, shift handover period, and team skill level measures. To note, we only had the shift handover period and the teamskill level measures for the NSCC operators. However, we kept all these variables in the regression models to predict both NSCC operation (T1 and T3) and SPCC operation (T2) for two reasons. First, the operation of the NSCC operators may have certain after effects on the SPCC operators’ work. In this way, controlling for certain NSCC properties can rule out alternative explanations. Second, it is easy to compare the variable contributions across different models by using the same independent variables. The results of the regression analysis are shown in Table 1.

**TABLE 1:** The regression analyses of all three time measures.

Independent variables	T1 (Judgment of navigation service failure, NSCC)	T2 (Satellite platform diagnosis and recovery, SPCC)	T3 (Navigation service system re-initiation, NSCC)
(1) Task complexity	0.34***	0.46***	0.33***
(2) Shift handover period (NSCC)^+^	0.02	0.23	0.35***
(3) Average team skill level (NSCC)	0.09	–0.08	–0.08
(4) Highest team skill level (NSCC)	−0.27**	0.02	−0.32***
**Time of day**			
(5) 0:00–2:00	0.02	0.27*	0.15
(6) 2:00–4:00	–0.04	0.30*	0.09
(7) 4:00–6:00	–0.05	0.18	0.14
(8) 6:00–8:00	–0.11	0.14	0.11
(9) 8:00–10:00^++^	—	—	—
(10) 10:00–12:00	–0.14	0.08	0.13
(11) 12:00–14:00	0.00	0.03	0.11
(12) 14:00–16:00	–0.15	0.12	0.06
(13) 16:00–18:00	–0.15	0.04	0.08
(14) 18:00–20:00	0.08	0.18	0.15
(15) 20:00–22:00	–0.13	0.03	0.03
(16) 22:00–24:00	–0.06	0.04	0.12
*R*^2^	0.23***	0.35***	0.27***
*F*	2.933	5.024	3.341

*Significant at 0.05 level, **Significant at 0.01 level, ***Significant at 0.001 level. +Shift handover Period was coded as 1 when the time is 30 min within shift handover, as 0 if it is out of this period; ++reference category.

In predicting T1, the whole model was significant, and all variables accounted for 23% of the total variance of this initial judgment time. Task complexity (β = 0.34, *p* < 0.001) and the highest team skill level (β = −0.27, *p* < 0.01) turned out to be significant predictors. More time was spent on stage 1 if the task was more complex and the skill of the most adept operator in the team was lower.

In predicting T2, the whole model was significant, and all variables accounted for 35% of the total variance of the satellite platform diagnosis and recovery time. Task complexity (β = 0.46, *p* < 0.001), time periods 0:00–2:00 (β = −0.27, *p* < 0.01) and 2:00–4:00 (β = −0.30, *p* < 0.01) turned out to be significant predictors. More time was spent on stage 2 if the task was more complex and the time was between midnight and 4 a.m.

In predicting T3, the whole model was significant, and all variables accounted for 27% of the total variance. Task complexity (β = 0.33, *p* < 0.001), the shift handover period (β = 0.35, *p* < 0.001) and the highest team skill level (β = −0.32, *p* < 0.001) turned out to be significant predictors. More time was spent on stage 3 if the task was more complex, the incidents happened during the shift handover period, and the skill of the most adept operator in the team was lower.

## Discussion

This study sought to examine how task, environment, and team characteristics jointly influence the system recovering process after equipment failure of the Beidou satellite navigation system using real event records. Three stages (judgment of navigation service failure, satellite platform diagnosis and recovery and navigation service re-initiation) were identified, and their execution time was analyzed. Several findings are worth discussion.

### Task Complexity Impacted to Operator’s Response Time

The results demonstrated that task complexity turned out to be the most significant predictor of the task completion time of all three stages. This is not surprising given the fact that the difficulty to use the signal directly and the numbers of failed modules are linked closely with both mental (diagnosis) and physical (execution) operations. When the satellite is out of direct signal receiving areas, operators need to use multiple sources of information to figure out its current situation. Also, as compared with the situation in which only one module fails, more operations are needed when two modules fail. It is interesting that the relationship between task complexity and satellite platform recovering time (T2, β = 0.46, *p* < 0.000) was closer as compared to its relationships with navigation service failure diagnosis time (T1, β = 0.34, *p* < 0.001) and navigation service system re-initiation time (T3, β = 0.33, *p* < 0.001). To note, in performing the first and the final stages of operation, operators only need to make a one-way communication with the satellite, following a single thread without alternative operations, i.e., either using the data sent from the satellite (the first stage) or issuing a command to the satellite (the final stage). Even the steps to confirm whether the navigation signal is normal are routine operations. However, in performing the second stage of operation, controllers (at another center) are more involved with two-way communication: they have to send commands to the satellite to shut down or restart certain equipment that may account for the problem and wait for the responses from the satellite to see whether it works. This process may repeat back and forth several times until they successfully locate the real problem and find ways to handle it. In this way, the increase in task complexity may make this process more complicated as it requires relatively more operational steps.

### The Failures Near Shifting Time Require Longer Re-initiate Time

The time to re-initiate the navigation service system (T3) was prolonged when the event happened during the shift handover period. In this case, the two shifts have much information to transfer during the handover, which may affect the operation of the third stage. However, the initial judgment time (T1) was not influenced by this variable. The reason might be that since the first stage of operation requires a fast response which generally lasts for quite a short period of time (*M* = 3.02 min, *SD* = 2.55), even if there are many items to be included in the handover, operators may prioritize dealing with this short but urgent task. However, when it requires a long-term operation such as the third stage work (*M* = 52.71 min, *SD* = 29.97), operators in a handover period may not be able to invest all their resources to deal with such operation. Since the second stage of the operation is performed by operators at another center whose shift handover period was not revealed, it is not surprising that this variable did not influence T2.

### Circadian Impacted to Human Performance

The time of day an event occurred had an influence on T2, but not on T1 and T3. The satellite platform recovery time was significantly longer when the incidents happened between 0:00 and 4:00, which is in accordance with previous findings that people have lowest physiological arousal and mental and physical readiness at this time (Colquhoun, 1972). The reasons why T1 and T3 were not influenced by time of day can be many. First, the initial diagnosis can be made very fast after the very loud alert signal, which may undermine the effects of reduced arousal, so the completion time would not be influenced by this variable. For the operators doing the third stage task (system reinitiating), since they have been awakened previously, they can get themselves prepared when waiting for the second stage to be finished. In this period, they can arrange their time to take some rest or drink coffee so they can get vitalized. As a result, although this stage lasts quite long, it is also not influenced by time of day.

### The Formation of Team Experience Influenced Diagnosis

The highest level of team expertise had a significant effect on T1 and T3. This result indicates that the performance of a team is significantly influenced by the team member with the most professional background. Nevertheless, the average team skill level was not found to have any significant influences. This finding can be explained in two different ways. First, it is possible that the task allocation in the satellite control center is not very interdependent and the work of any specific operation is only assigned to the operator who has the most relevant skill. In this way, if he/she fails, the whole process can be undermined. Second, it is also possible that even if the team members coordinate intensively in this process, the knowledge and skills to perform the diagnosis and operation are not additive. All operators will follow the instructions of the most adept operators in their shift team, and his/her failure may result in prolonged reaction time of the whole team. Since we did not collect the team composition data from the SPCC, it is not surprising that the team skill levels did not influence on T2 (the On-board Diagnosis Time performed by SPCC operators).

### Limitations

Several limitations must be mentioned before concluding. In the first place, the event log file we used in this study, albeit very real, had certain drawbacks. For example, since all the times were recorded only down to minute (also see the method section), the regression model for T1 might be comparatively less reliable than the models established for T2 and T3 because the measurement error occupies a larger ratio for the shorter time intervals. Future studies may benefit from using a more accurate recording system. Second, we only made analyses based on the most common incidents (the failure of Instrument N). Although other types of failure have a lot in common with the incidents we analyzed, it can be further explored in future studies whether the human factor variables identified in this study can be generalized to the responses to other types of incidents. Besides, we only collected the data from the NSCC but failed to collect the team composition and the shift schedule of SPCC operators. Although we correctly found T2 (the time performed by the SPCC operators) could not be predicted by the team composition and the shift schedule of NSCC operators, more explanatory power can be added if we could use more relevant information from the SPCC operators.

## Conclusion

This study attempts to provide an initial understanding of how task, environmental, and team characteristics jointly influence the fault handling process of the Beidou satellite navigation system. This study made an analysis based on real incident records. We found many human factor variables predictive of the completion time for an instrument failure recovery, which could explain a certain amount of variance (23–35%). These findings offer some implications for the Beidou satellite navigation system. Above all, since task complexity is the strongest predictor of the fault handling time, efforts should be paid to lower it in multiple aspects, such as instrument optimization, operation simplification, and interface usability. Second, the SPCC is suggested to attach more importance to the time period 0:00–4:00, during which the satellite platform recovery time is prolonged. A possible solution is to add operators, especially those energetic at night. Though certain effects of other environment and team characteristics have not been confirmed for the SPCC, it can be pursued further in the future. Furthermore, given the significance of the team’s highest skill level for the recovery process, making sure there is at least one competent operator in each team will be helpful. For researchers, future studies may benefit from using a more accurate recording system of both the events and the individuals. For practitioners, it is worth thinking whether the process can be improved by removing certain obstacles that may undermine human performance.

## Data Availability Statement

The datasets for this article are not publicly available because the raw data contains key information for the safe operation for the complex satellite navigation system. Requests to access the datasets should be directed to MW, wu_mo@sohu.com.

## Author Contributions

MW, LZ, and JZ designed the study and wrote the manuscript. NL and JZ conducted the statistical analysis. MW prepared figures and tables. NL, LW, and W-CL edited the manuscript. All authors reviewed the manuscript.

## Conflict of Interest

The authors declare that the research was conducted in the absence of any commercial or financial relationships that could be construed as a potential conflict of interest.

## Footnotes

1 For confidential reasons, the actual name of the Instrument N was not revealed.

## References

[B1] Bell S. T. (2007). Deep-level composition variables as predictors of team performance: a meta-analysis. *J. Appl. Psychol.* 92 595–615. 10.1037/0021-9010.92.3.595 17484544

[B2] Borst C. Bijsterbosch V. A. van Paassen M. M. Mulder M. (2017). Ecological interface design: supporting fault diagnosis of automated advice in a supervisory air traffic control task. *Cogn. Technol. Work* 19 545–560. 10.1007/s10111-017-0438-y

[B3] Brazier A. Sedgwick M. (2011). *The Link Between Safety and Shift Handover.* Aberdeen: Offshore Europe.

[B4] Campbell S. S. (1992). “Effects of sleep and circadian rhythms on performance,” in *Handbook of Human Performanceeds, State and Trait*, Vol. 3 eds Smith A. P. Jones D. (London: Academic Press).

[B5] Charlton S. G. (1992). Establishing human factors criteria for space control systems. *Hum. Factors* 34 485–501. 10.1177/001872089203400410 11871455

[B6] Chen K. Ge M. Babeyko A. Li X. Diao F. Tu R. (2016). Retrieving real-time co-seismic displacements using GPS/GLONASS: a preliminary report from the September 2015Mw8.3 Illapel earthquake in Chile. *Geophys. J. Int.* 206 941–953. 10.1093/gji/ggw190

[B7] Colquhoun W.P. (ed.) (1972). *Aspects of Human Efficiency: Diurnal Rhythms and Loss of Sleep.* London: English Universities Press.

[B8] Cymek D. H. (2018). Redundant automation monitoring: four eyes don’t see more than two, if everyone turns a blind eye. *Hum. Factors* 60 902–921. 10.1177/0018720818781192 29939767

[B9] Devine D. J. Philips J. L. (2001). Do smarter teams do better. *Small Group Res.* 32 507–532. 10.1177/104649640103200501 15029794

[B10] Dongen H. P. A. V. Dinges D. F. (2000). “Circadian rhythms in fatigue, alertness, and performance,” in *Principles and Practice of Sleep Medicine*, 3rd Edn, eds Kryger M. H. Roth T. Dement W. C. (Philadelphia, PA: W.B. Saunders), 391–399.

[B11] Doytchev D. E. Szwillus G. (2009). Combining task analysis and fault tree analysis for accident and incident analysis: a case study from Bulgaria. *Accid. Anal. Prev.* 41 1172–1179. 10.1016/j.aap.2008.07.014 19819365

[B12] Folkard S. (1983). “Diurnal variation,” in *Stress and Fatigue in Human Performance*, ed. Hockey G. R. J. (Chichester: Wiley).

[B13] Funke G. J. Warm J. S. Baldwin C. L. Garcia A. Funke M. E. Dillard M. B. (2016). The independence and interdependence of coacting observers in regard to performance efficiency, workload, and stress in a vigilance task. *Hum. Factors* 58 915–926. 10.1177/0018720816646657 27150529

[B14] Gonzalez C. Vanyukov P. Martin M. K. (2005). The use of microworlds to study dynamic decision making. *Comput. Human Behav.* 21 273–286. 10.1016/j.chb.2004.02.014

[B15] Gordon R. P. E. (1998). The contribution of human factors to accidents in the offshore oil industry. *Reliab. Eng. Syst. Saf.* 61 95–108. 10.1590/0102-311X00034617 29617478

[B16] Ilgen D. R. Hollenbeck J. R. Johnson M. Jundt D. (2005). Teams in organizations: from input-process-output models to imoi models. *Annu. Rev. Psychol.* 56 517–543. 10.1146/annurev.psych.56.091103.070250 15709945

[B17] Kearney E. Gebert D. Voelpel S. C. (2009). When and how diversity benefits teams: the importance of team members’ need for cognition. *Acad. Manag. J.* 52 581–598. 10.5465/amj.2009.41331431

[B18] Kontogiannis T. Leopoulos V. Marmaras N. (2000). A comparison of accident analysis techniques for safety-critical man–machine systems. *Int. J. Ind. Ergon.* 25 327–347. 10.1016/s0169-8141(99)00022-0

[B19] Kuhr J. W. Tobias L. W. (1974). “Human factors engineering aspects of satellite communication systems,” in *Proceedings of the Human Factors Society 18th Annual Meeting*, Huntsville, AL, 604–623. 10.1177/154193127401800521

[B20] Langer M. Braithwaite G. R. (2016). The development and deployment of a maintenance operations safety survey. *Hum. Factors* 58 986–1006. 10.1177/0018720816656085 27411354 PMC5052696

[B21] Leveson I. (2015). “The economic value of GPS: preliminary assessment,” in *Proceedings of the National Space-Based Positioning, Navigation and Timing Advisory Board Meeting*, Boulder, CO.

[B22] Littlepage G. E. Hein M. B. Moffett R. G. Craig P. A. Georgiou A. M. (2016). Team training for dynamic cross-functional teams in aviation. *Hum. Factors* 58 1275–1288. 10.1177/0018720816665200 27549389

[B23] Liu P. Li Z. (2011). Toward understanding the relationship between task complexity and task performance. *Int. Des. Glob. Dev.* 6775 192–200. 10.1007/978-3-642-21660-2_22

[B24] Liu P. Li Z. (2012). Task complexity: a review and conceptualization framework. *Int. J. Ind. Ergon.* 42 553–568. 10.1016/j.ergon.2012.09.001

[B25] Martin K. McLeod E. Périard J. Rattray B. Keegan R. Pyne D. B. (2019). The impact of environmental stress on cognitive performance: a systematic review. *Hum. Factors* 61 1205–1246. 10.1177/0018720819839817 31002273

[B26] Mathieu J. E. Tannenbaum S. I. Donsbach J. S. Alliger G. M. (2013). A review and integration of team composition models. *J. Manag.* 40 130–160. 10.1177/0149206313503014

[B27] Mitchell C. M. (1982). “Human factors dimensions in the evolution of increasingly automated control rooms for near-earth satellites,” in *Proceedings of the 26th Annual Meeting: Human Factors and Ergonomics Society, Seattle, WA* (Santa Monica, CA: Human Factors Society), 155–159. 10.1177/154193128202600213

[B28] Molloy R. Parasuraman R. (1996). Monitoring an automated system for a single failure: vigilance and task complexity effects. *Hum. Factors* 38 311–322. 10.1177/001872089606380211 11536753

[B29] Monk T. Buysse D. Reynolds C. III Berga S. Jarrett D. Begley A. (1997). Circadian rhythms in human performance and mood under constant conditions. *J. Sleep Res.* 6 9–18. 10.1046/j.1365-2869.1997.00023.x 9125694

[B30] Mumaw R. J. Roth E. M. Vicente K. J. Burns C. M. (2000). There Is more to monitoring a nuclear power plant than meets the eye. *Hum. Factors* 42 36–55. 10917145 10.1518/001872000779656651

[B31] Neville K. Owens J. M. Eitelman S. M. Barba C. A. Ennis J. Hafich A. (2003). “Designing work-centered performance support solutions for operators of a complex and evolving system,” in *Proceedings of the Human Factors and Ergonomics Society 47th Annual Meeting* (Santa Monica, CA: HFES), 463–467. 10.1177/154193120304700345

[B32] Pepinsky P. N. Pepinsky H. B. Pavlik W. B. (1960). The effects of task complexity and time pressure upon team productivity. *J. Appl. Psychol.* 44 34–38. 10.1037/h0039877

[B33] Pezzolesi C. Manser T. Schifano F. Kostrzewski A. Pickles J. Harriet N. (2012). Human factors in clinical handover: development and testing of a “handover performance tool” for doctors’ shift handovers. *Int. J. Qual. Health Care* 25 58–65. 10.1093/intqhc/mzs076 23220763

[B34] Pezzolesi C. Schifano F. Pickles J. Randell W. Hussain Z. Muir H. (2010). Clinical handover incident reporting in one UK general hospital. *Int. J. Qual. Health Care* 22 396–401. 10.1093/intqhc/mzq048 20709704

[B35] Resick C. J. Dickson M. W. Mitchelson J. K. Allison L. K. Clark M. A. (2010). Team composition, cognition, and effectiveness: examining mental model similarity and accuracy. *Group Dyn.* 14 174–191. 10.1037/a0018444

[B36] Riesenberg L. A. (2012). Shift-to-shift handoff research: where do we go from here? *J. Grad. Med. Educ.* 4 4–8. 10.4300/jgme-d-11-00308.1 23451298 PMC3312531

[B37] Roberts A. P. J. Stanton N. A. Fay D. (2017). Land ahoy! Understanding submarine command and control during the completion of inshore operations. *Hum. Factors* 59 1263–1288. 10.1177/0018720817731678 28982016

[B38] Sebok A. Wickens C. D. (2016). Implementing lumberjacks and black swans into model-based tools to support human–automation interaction. *Hum. Factors* 59 189–203. 10.1177/0018720816665201 27591210

[B39] Siemsen I. M. D. Madsen M. D. Pedersen L. F. Michaelsen L. Pedersen A. V. Andersen H. B. (2012). Factors that impact on the safety of patient handovers: an interview study. *Scand. J. f Public Health* 40 439–448. 10.1177/1403494812453889 22798283

[B40] Tan H. Ng T. B. Mak B. W. (2002). The effects of task complexity on auditors’ performance: the impact of accountability and knowledge. *Auditing J. Pract. Theory* 21 81–95. 10.2308/aud.2002.21.2.81

[B41] Trapsilawati F. Wickens C. D. Qu X. Chen C.-H. (2016). Benefits of imperfect conflict resolution advisory aids for future air traffic control. *Hum. Factors* 58 1007–1019. 10.1177/0018720816655941 27422153

[B42] Van Balen P. M. Mitchell C. M. (1983). Development of a human factors methodology for nasa-goddard space flight center. *Proc. Hum. Factors Ergon. Soc. Annu. Meet.* 27 980–984. 10.1177/154193128302701209

[B43] Vicente K. J. Mumaw R. J. Roth E. M. (2004). Operator monitoring in a complex dynamic work environment: a qualitative cognitive model based on field observations. *Theor. Issues Ergon. Sci.* 5 359–384. 10.1080/14039220412331298929

[B44] Vicente K. J. Roth E. M. Mumaw R. J. (2001). How do operators monitor a complex, dynamic work domain? The impact of control room technology. *Int. J. Hum. Comput. Stud.* 54 831–856. 10.1006/ijhc.2001.0463

[B45] Wang Y. Zhao X. Pang C. Feng B. Tong H. Zhang L. (2019). BDS and GPS stand-alone and integrated attitude dilution of precision definition and comparison. *Adv. Space Res.* 63 2972–2981. 10.1016/j.asr.2017.11.032

[B46] Wickens C. D. Rice S. Keller D. Hutchins S. Hughes J. Clayton K. (2009). false alerts in air traffic control conflict alerting system: is there a “Cry Wolf” effect? *Hum. Factors* 51 446–462. 10.1177/0018720809344720 19899356

[B47] Wood R. E. (1986). Task complexity: definition of the construct. *Organ. Hum. Decis. Process.* 37 60–82. 10.1016/0749-5978(86)90044-0

[B48] Zhang J. Li Y. Wu C. (2013). The Influence of Individual and Team cognitive ability on operators’ task and safety performance: a multilevel field study in nuclear power plants. *PLoS One* 8:e84528. 10.1371/journal.pone.0084528 24391964 PMC3877292

